# A Retrospective Comparison of Artificial Intelligence and the Orthopaedic Multi-disciplinary Team in the Management of Intracapsular Neck of Femur Fractures

**DOI:** 10.7759/cureus.94699

**Published:** 2025-10-16

**Authors:** Matthew K Emmerson, Ryan Hillier-Smith, Amar Malhas

**Affiliations:** 1 Orthopaedics, Royal Berkshire NHS Foundation Trust, Reading, GBR

**Keywords:** artifical intelligence, clinical-decision making, elderly hip fractures, elderly trauma, orthopaedic surgery

## Abstract

Introduction

Artificial intelligence (AI) tools, such as ChatGPT, could potentially support junior clinicians in making initial operative decisions for hip fractures. However, the safety and reliability of such use are uncertain. This study compared ChatGPT’s management recommendations for patients with intracapsular neck of femur (NOF) fractures to decisions made by orthopaedic consultants and evaluated which patient factors influenced those recommendations.

Methods

We identified a retrospective cohort of patients admitted with an intracapsular NOF fracture over an 18-week period to a United Kingdom District General Hospital. We collected patients’ age, sex, comorbidities, mobility status, and the 4 A’s Test (4AT) score. De-identified data were entered into ChatGPT with instructions to recommend management based on National Institute for Health and Care Excellence guidance; ChatGPT’s recommendations were compared with the operation recorded at the Trauma Meeting. When ChatGPT and consultants disagreed, we provided ChatGPT with the consultant's decision and rationale and recorded the revised recommendation. We then validated ChatGPT on a separate anonymized patient set. We used Cohen’s Kappa to assess agreement and applied multinomial and binomial logistic regression and two-proportion z-tests to assess significance.

Results

One hundred five patients were included in the primary cohort, and 30 in the validation cohort. Initial agreement between ChatGPT and consultants was low [κ = 0.03, 95% confidence interval (CI) - 0.11 to 0.19, p = 0.70]. After in-session adjustment, agreement rose (κ = 0.93, 95% CI 0.84 - 1.00, p < 0.001), a statistically significant improvement (z = 10.3, p < 0.001). The only post-adjustment factor that significantly influenced ChatGPT’s recommendations was age, showing that increasing age was associated with a reduced likelihood of receiving total hip replacement rather than hemiarthroplasty (odds ratio = 0.81, 95% CI 0.70-0.94; p = 0.006). In the validation cohort, agreement fell (κ = 0.29, 95% CI -0.04 to 0.60, p = 0.06) and did not significantly differ from the initial agreement (z = -1.51, p = 0.13).

Conclusion

ChatGPT did not reliably replicate orthopaedic consultant decision-making for intracapsular NOF fractures. Although in-session adjustments produced high superficial concordance, this effect did not generalize to an independent dataset. The model’s tendency to conform to user prompts risks creating false confidence in its outputs. Clinically focused, validated AI systems require further evaluation before they can safely augment operative decision-making.

## Introduction

Hip fractures are a common orthopaedic presentation. On-call orthopaedic trainees commonly admit and prepare patients for surgery. Teams typically use protocols, textbooks, and online resources to guide initial surgical decisions pending the operating consultant’s definitive decision at the morning multidisciplinary meeting. Uncertainty can lead to patients being prepared for inappropriate procedures or to duplication of work, delaying operations, delays that are associated with higher mortality, longer hospital stays, and poorer postoperative outcomes [1,2]. Currently in the United Kingdom (UK), only 56% of hip fracture patients receive an operation within 36 hours [3]. Thus, resources that support on-call trainee decision-making could increase adherence to best-practice tariffs for hip fractures.

Artificial intelligence (AI) programs, such as ChatGPT, are being increasingly investigated in other medical specialties as aids to clinician decision-making for triage [4], diagnosis [5], and further management [6]. In orthopaedics, applications to date have been limited to answering patients’ questions about operations [7,8] or making management decisions from imaging alone [9]; no studies have assessed whether AI can support orthopaedic management decisions that incorporate broader patient context. Given clear guidance from the National Institute for Health and Care Excellence (NICE) on hip fracture management [10], ChatGPT could serve as a reference for more junior clinicians. However, little work has established whether this can be done safely and reliably.

General-purpose large language models (LLMs), such as ChatGPT, are not medical decision-support systems and are not trained for clinical use. Known limitations include hallucinations [11,12] and limited explainability [13], which hinder reliable, transparent clinical decision-making. Furthermore, LLMs have not been validated for clinical use and remain in early stages of evaluation for medical applications [4-6]. A common misconception concerns LLMs’ “trainability.” Unlike some machine-learning models, ChatGPT cannot be trained during a user session; it was trained on large corpora of publicly available data [14]. Consequently, errors that arise in a session cannot be corrected by in-session retraining-users can only attempt to steer outputs through prompt adjustments. Therefore, investigations (including this study) that compare clinician and LLM decision-making remain preliminary.

This study aimed to compare ChatGPT’s management recommendations for patients with intracapsular neck of femur (NOF) fractures to decisions made by orthopaedic consultants. We assessed agreement between ChatGPT and consultants and examined the relative importance of patient-specific factors in shaping management decisions. This study aims to guide clinicians on the current gaps in AI validation for surgical decision-making.

## Materials and methods

Study design and data extraction

We performed a retrospective cohort study of patients admitted to a District General Hospital (DGH) in the UK with an intracapsular NOF fracture who were entered into the National Hip Fracture Database [15] over 18 weeks (01/10/2023 to 10/02/2024). We extracted patients’ age, sex, comorbidities, mobility status, 4 A's test (4AT) score (a delirium screening tool) [16,17], and the operation performed from the electronic patient record, specifically the orthopaedic clerking document. Comorbidities were taken from both the clerking document and the General Practitioner records; when discrepancies occurred, the orthopaedic clerking document took precedence. The data were anonymised and entered into a spreadsheet. At this DGH, the hip operation is determined at a Trauma Meeting by the orthopaedic multidisciplinary team (MDT), where orthopaedic consultants, anaesthetists, orthopaedic trainees, and trauma nurse practitioners discuss each patient. The study was registered with the local Quality Governance and Ethics board (Royal Berkshire Hospital; reference number N5352). All patient data were anonymised and further de-identified by removing any dates and suppressing rare diseases. We opted out of allowing inputs to be used for external model training when data were entered into ChatGPT [18]. As a result, no data flowed outside the temporarily closed chat system created on the ChatGPT web interface. All data were deleted within 30 days of chat creation, ensuring no patient information was stored on external servers. Access to the temporary chat was restricted by password protection. Consequently, individual consent was not obtained. All methods complied with local data-protection regulations.

First round of ChatGPT responses

We generated prompts in an Excel spreadsheet with columns titled “ChatGPT Prompt” and “Answer.” An example prompt based on a fictional patient is shown below (the full set of prompt options appears in Appendix 1).

“A 72-year-old female presents to the emergency department with an intracapsular neck of femur fracture. Their co-morbidities include Dementia, type 1 diabetes. They walk with a frame. Their 4AT score is 7. Using the most recent NICE guidelines on Hip fracture management, what operation should they have to fix their intracapsular neck of femur fracture?”

We piloted the prompts on a sample of 10 patients and then applied the same template to every patient, modifying only the patient-specific details. We uploaded the spreadsheet to ChatGPT via the web interface on the ChatGPT Plus plan and used the GPT-4o model throughout the study; no plug-ins were used, and the only setting change was opting out of inputs for external model training [19]. The first-round responses were generated on 22/1/25 at 18:02 GMT using the instruction: “Please respond to the prompts in the ‘ChatGPT Prompt’ column with the name of the surgery recommended in the ‘Answer’ column of the attached Excel spreadsheet.” We compared ChatGPT’s recommendations with the operations recorded as decided at the Trauma Meeting to assess agreement.

Second round of ChatGPT responses

For the patients in which disagreement between ChatGPT and the Trauma Meeting decision occurred, we extracted the Trauma Meeting rationale from the electronic notes; if no rationale was documented, members of the research team discussed the case with orthopaedic consultants to establish the likely rationale. We then created a second prompt that included the original ChatGPT response, the Trauma Meeting outcome, and the documented rationale. An example prompt is shown below, and further options are provided in Appendix 2.

“A 76-year-old female presents to the emergency department with an intracapsular neck of femur fracture. Their comorbidities include Hypothyroidism. They are independent with walking. Their 4AT score is 0. ChatGPT previously recommended the patient should have a Hemiarthroplasty to fix their intracapsular neck of femur fracture according to NICE guidelines; however, several orthopaedic consultants disagree with the decision and instead think the patient should be treated with Total hip replacement. This is because they believe the patient is fit and mobile. Using the most recent NICE guidelines on Hip fracture management and the information given, would you change your management and follow the consultants' management suggestion, or would you stick with the previous ChatGPT management suggestion?”

We submitted each revised prompt to the same ChatGPT chat session on 31/1/25 at 18:34 GMT and recorded ChatGPT’s second-round recommendations and the model’s stated rationale in the spreadsheet. We then assessed agreement between the second ChatGPT responses and the Trauma Meeting decisions.

Clinical validation attempt

To assess reproducibility, we identified a separate validation cohort of patients admitted with an intracapsular NOF fracture and entered into the National Hip Fracture Database over one month (22/4/24 to 22/5/24). We extracted and anonymised the same variables as for the primary cohort and generated prompts using the identical template and spreadsheet process described above. The validation prompts were submitted to ChatGPT, and the responses were generated on 5/8/25 at 11:52 BST.

Statistical analysis

We performed statistical analyses with Wizard 2 (Evan Miller, Chicago, USA) and Jamovi (The jamovi project, Sydney, Australia) [20-23]. We used Cohen’s kappa (κ) to evaluate agreement between ChatGPT and Trauma Meeting responses in the first, second, and validation rounds. Confusion matrices provided observed and expected agreement. We estimated standard errors and 95% confidence intervals (CIs) for κ using nonparametric bootstrapping and used z tests to assess whether κ differed from zero and whether κ changed between rounds. We used multinomial logistic regression to assess associations between specific factors and Trauma Meeting and ChatGPT responses in the second round. We used binomial logistic regression to assess associations between specific factors and ChatGPT responses in the first and validation rounds because ChatGPT offered only two operative options in those rounds. Because of small cell counts, we collapsed dynamic hip screw, cannulated hip screw, and no operation into a single category, “Fixation/No Operation.” We also collapsed mobility into “independent with walking,” “walking with a stick,” and “less mobile than walking with a stick.” For logistic regression, the reference categories were hemiarthroplasty, female, and independent with walking. We considered p < 0.05 statistically significant.

## Results

Overall demographics

One hundred nine patients were initially identified. Two patients were excluded because they sustained extracapsular neck of femur fractures, and two were excluded because their notes could not be located. The final cohort comprised 105 patients. Sixty-seven patients were female (63.8%). The mean number of preoperative comorbidities was 4.29 [standard deviation (SD) = 2.34], and the mean preoperative 4AT score was 3.01 (SD = 4.17). Preoperatively, 44 patients were independent with walking (41.9%), 26 walked with a stick (24.8%), 32 walked with a frame (30.5%), two mobilised with a wheelchair (1.9%), and one patient was immobile (1.0%).

Operation decisions, agreement, and disagreement

The operations selected at Trauma Meetings and the procedures recommended by ChatGPT in the first and second rounds are summarised in Table 1. In the first round, agreement between ChatGPT and the Trauma Meeting was poor (Cohen’s kappa [κ] = 0.03; 95% CI, −0.11 to 0.19; p = 0.70), indicating no statistically significant agreement. Table 2 shows the first-round confusion matrix. Table 3 reports reasons for disagreement; the most common documented rationale for consultant decision-making that differed from ChatGPT was that the patient was “fit and mobile” (10 cases).

**Table 1:** Management of intracapsular neck of femur fractures as determined by Trauma Meeting and ChatGPT in the first and second round of prompts

Operation	Trauma Meeting Decision, n patients	First Round ChatGPT Recommendation, n patients	Second Round ChatGPT Recommendation, n patients
Hemiarthroplasty	79	89	81
Total Hip Replacement	13	16	12
Cannulated Hip Screw	5	0	6
Dynamic Hip Screw	3	0	2
No Operation	5	0	4

**Table 2:** Confusion matrix demonstrating operative management decisions made by Trauma Meeting and first round of ChatGPT responses

Decision	ChatGPT Hemiarthroplasty	ChatGPT Total Hip Replacement	ChatGPT Cannulated Hip Screw	ChatGPT Dynamic Hip Screw	ChatGPT No Operation
Trauma Meeting Hemiarthroplasty	67	12	0	0	0
Trauma Meeting Total Hip Replacement	10	3	0	0	0
Trauma Meeting Cannulated Hip Screw	5	0	0	0	0
Trauma Meeting Dynamic Hip Screw	3	0	0	0	0
Trauma Meeting No Operation	4	1	0	0	0

**Table 3:** Reasons for disagreement between Trauma Meeting and ChatGPT

Reason for Trauma Meeting Decision	ChatGPT Disagreement with Trauma Meeting, n patients (%)
Fit and mobile	10 (28.6%)
Extensive significant past medical history	4 (11.4%)
Not significantly displaced fracture	4 (11.4%)
Poor mobility and extensive, significant past medical history	3 (8.6%)
Confused/dementia	3 (8.6%)
Clinical trial	2 (5.7%)
Short anaesthetic duration was required	2 (5.7%)
Poor mobility	2 (5.7%)
Poor soft tissue coverage	1 (2.9%)
Patient choice	1 (2.9%)
Sarcoma found on the fractured hip	1 (2.9%)
Extensive existing metalwork and prosthesis around the fractured hip/leg	1 (2.9%)
End of life	1 (2.9%)

Trauma Meeting decision factors and first-round ChatGPT associations

Tables 4, 5, 6 show the multinomial logistic regression analysis of Trauma Meeting decisions. The only statistically significant finding was that increasing age was associated with a decreasing likelihood of receiving a total hip replacement (THR) compared with hemiarthroplasty [odds ratio (OR) = 0.80; 95% CI, 0.70-0.92; p = 0.002].

**Table 4:** Multinomial logistic regression—model fit measures (N = 105) Models estimated using a sample size of N = 105. Abbreviation: AIC, Akaike Information Criterion.

Model	Deviance	AIC	R² (McFadden)
1	104	132	0.320

**Table 5:** Multinomial logistic regression—Fixation/No operation vs Hemiarthroplasty (reference outcome: Hemiarthroplasty) 4AT: 4 A’s Test (delirium screen); SE: standard error; CI: confidence interval.

Predictor (reference in parentheses)	Estimate	SE	z	P-Value	Odds ratio	95% CI lower	95% CI upper
Intercept	2.25822	3.4575	0.6531	0.514	9.5661	0.01091	8390.351
Age (per year)	-0.04164	0.0428	-0.9721	0.331	0.9592	0.88198	1.043
Number of comorbidities	0.03864	0.1463	0.2641	0.792	1.0394	0.78025	1.385
4AT score	-0.35108	0.1891	-1.8567	0.063	0.7039	0.48594	1.020
Male (female)	0.06457	0.6912	0.0934	0.926	1.0667	0.27524	4.134
Less mobile than walking with a stick (independent with walking)	-0.07929	0.8644	-0.0917	0.927	0.9238	0.16974	5.027
Walk with a stick (independent with walking)	-0.49197	0.7980	-0.6165	0.538	0.6114	0.12796	2.922

**Table 6:** Multinomial logistic regression—Total Hip Replacement vs Hemiarthroplasty (reference outcome: Hemiarthroplasty) 4AT: 4 A’s Test (delirium screen); SE: standard error; CI: confidence interval.

Predictor (reference in parentheses)	Estimate	SE	z	P-Value	Odds ratio	95% CI lower	95% CI upper
Intercept	17.11474	5.4527	3.1388	0.002	2.71e+7	618.77005	1.19e+12
Age (per year)	-0.21737	0.0711	-3.0568	0.002	0.8046	0.69995	0.925
Number of comorbidities	0.00908	0.2395	0.0379	0.970	1.0091	0.63111	1.614
4AT score	-1.20052	0.8949	-1.3416	0.180	0.3010	0.05211	1.739
Male (female)	-1.20664	0.9894	-1.2196	0.223	0.2992	0.04303	2.080
Less mobile than walking with a stick (independent with walking)	-2.45491	1.5741	-1.5595	0.119	0.0859	0.00393	1.878
Walk with a stick (independent with walking)	-1.94743	1.2543	-1.5526	0.121	0.1426	0.01221	1.667

Tables 7, 8 show the binomial logistic regression analysis for the first round of ChatGPT responses. One significant finding was that, compared with patients receiving hemiarthroplasty, a greater number of comorbidities was associated with increased odds of undergoing THR (OR = 1.46; 95% CI, 1.09-1.94; p = 0.01). Sex was also associated with the outcome (male vs female: OR = 0.07; 95% CI, 0.01-0.63; p = 0.02).

**Table 7:** Binomial logistic regression—model fit measures for round 1 ChatGPT decision (N = 105) Models estimated using a sample size of N = 105. AIC: Akaike information criterion.

Model	Deviance	AIC	R² (McFadden)
1	70.6	84.6	0.212

**Table 8:** Binomial logistic regression—predictors of round 1 ChatGPT decision (Total hip replacement vs Hemiarthroplasty) Estimates represent the log-odds of “ChatGPT decision = Total hip replacement” vs “ChatGPT decision = Hemiarthroplasty.”  4AT: 4 A’s Test (delirium screen); SE: standard error; CI: confidence interval.

Predictor (reference in parentheses)	Estimate	SE	z	P-Value	Odds ratio	95% CI lower	95% CI upper
Intercept	-2.91585	3.8609	-0.755	0.450	0.0542	2.80e-5	104.726
Age (per year)	0.00816	0.0479	0.170	0.865	1.0082	0.91777	1.108
Number of comorbidities	0.37614	0.1471	2.557	0.011	1.4567	1.09176	1.944
4AT score	-0.03258	0.0745	-0.437	0.662	0.9679	0.83636	1.120
Male (female)	-2.63007	1.1066	-2.377	0.017	0.0721	0.00824	0.631
Less mobile than walking with a stick (independent with walking)	-1.31253	0.7818	-1.679	0.093	0.2691	0.05814	1.246
Walk with a stick (independent with walking)	-1.18605	0.9074	-1.307	0.191	0.3054	0.05158	1.808

Second-round ChatGPT agreement and decision factors

Among the subset of patients for whom ChatGPT initially disagreed with the Trauma Meeting, the second-round responses achieved 91.4% agreement (32/35; 95% CI, 82% to 100%). Combining these resolved cases with the originally concordant cases produced an overall almost perfect agreement between ChatGPT and Trauma Meetings (κ = 0.93, 95% CI 0.84 - 1.00, p < 0.001). Table 9 shows the second-round confusion matrix. This improvement in agreement from the first to the second round was statistically significant (z = 10.3, p < 0.001).

**Table 9:** Confusion Matrix demonstrating operative management decisions made by Trauma Meeting and 2nd round of ChatGPT responses

Decision	ChatGPT Hemiarthroplasty	ChatGPT Total Hip Replacement	ChatGPT Cannulated Hip Screw	ChatGPT Dynamic Hip Screw	ChatGPT No Operation
Trauma Meeting Hemiarthroplasty	79	0	0	0	0
Trauma Meeting Total Hip Replacement	1	12	0	0	0
Trauma Meeting Cannulated Hip Screw	0	0	5	0	0
Trauma Meeting Dynamic Hip Screw	1	0	0	2	0
Trauma Meeting No Operation	0	0	1	0	4

Tables 10, 11, 12 show the multinomial logistic regression analysis of ChatGPT decisions in the second round. The one significant finding was that increasing age was associated with a lower likelihood of receiving THR compared with hemiarthroplasty (OR = 0.81; 95% CI, 0.70-0.94; p = 0.006).

**Table 10:** Multinomial logistic regression—model fit measures for round 2 ChatGPT decision (N = 105) Models estimated using a sample size of N = 105. AIC: Akaike information criterion.

Model	Deviance	AIC	R² (McFadden)
1	97.8	126	0.331

**Table 11:** Multinomial logistic regression—round 2 ChatGPT: Fixation/No operation vs Hemiarthroplasty (reference outcome: Hemiarthroplasty) 4AT: 4 A’s Test (delirium screen); SE: standard error; CI: confidence interval.

Predictor (reference in parentheses)	Estimate	SE	z	P-Value	Odds ratio	95% CI lower	95% CI upper
Intercept	1.3439	3.2967	0.4076	0.684	3.834	0.00599	2453.650
Age (per year)	-0.0292	0.0408	-0.7154	0.474	0.971	0.89654	1.052
Number of comorbidities	-0.0137	0.1525	-0.0898	0.928	0.986	0.73156	1.330
4AT score	-0.3231	0.1847	-1.7493	0.080	0.724	0.50404	1.040
Male (female)	0.1701	0.7099	0.2397	0.811	1.185	0.29487	4.766
Less mobile than walking with a stick (independent with walking)	-0.0686	0.8504	-0.0807	0.936	0.934	0.17634	4.944
Walk with a stick (independent with walking)	-0.9408	0.8920	-1.0548	0.292	0.390	0.06794	2.242

**Table 12:** Multinomial logistic regression—round 2 ChatGPT: Total hip replacement vs Hemiarthroplasty (reference outcome: Hemiarthroplasty) 4AT: 4 A’s Test (delirium screen); SE: standard error; CI: confidence interval; AIC: Akaike information criterion.

Predictor (reference in parentheses)	Estimate	SE	z	P-Value	Odds ratio	95% CI lower	95% CI upper
Intercept	16.8807	5.9443	2.8398	0.005	2.14e+7	186.81340	2.46e+12
Age (per year)	-0.2075	0.0753	-2.7567	0.006	0.813	0.70111	0.942
Number of comorbidities	-0.1384	0.2630	-0.5263	0.599	0.871	0.51998	1.458
4AT score	-0.7777	0.6647	-1.1699	0.242	0.459	0.12487	1.691
Male (female)	-1.7533	1.1333	-1.5471	0.122	0.173	0.01879	1.597
Less mobile than walking with a stick (independent with walking)	-13.8767	7.78e-4	-17837.1488	< 0.001	9.41e-7	9.39e-7	9.42e-7
Walk with a stick (independent with walking)	-1.9889	1.2491	-1.5923	0.111	0.137	0.01183	1.583

Validation cohort

Thirty new patients were included in the validation cohort. Twenty patients were female (66.7%). The mean number of preoperative comorbidities was 5.13 (SD = 2.52), and the mean preoperative 4AT score was 1.3 (SD = 3.17). Preoperatively, 12 patients were independent with walking (40.0%), four walked with a stick (13.3%), 10 walked with a frame (33.3%), three mobilised with a wheelchair (10.0%), and one was immobile (3.3%). Operations for the validation cohort are shown in Table 13.

**Table 13:** Comparing management of intracapsular neck of femur fractures as determined by Trauma Meeting and ChatGPT for the validation sample of patients

Operation	Trauma Meeting Decision, n patients	ChatGPT recommendation, n patients
Hemiarthroplasty	19	26
Total Hip Replacement	6	4
Cannulated Hip Screw	3	0
Dynamic Hip Screw	0	0
No Operation	2	0

In the validation round, ChatGPT showed fair agreement with the Trauma Meeting (κ = 0.29; 95% CI, −0.04 to 0.60), but this did not reach statistical significance (p = 0.06). Table 14 shows the confusion matrix for the validation cohort. This agreement did not differ significantly from the first-round agreement (z = −1.51; p = 0.13). The number of patients receiving each operation in the validation cohort was too small to perform logistic regression.

**Table 14:** Confusion matrix demonstrating operative management decisions made by Trauma Meeting and validation round of ChatGPT responses

Decision	ChatGPT Hemiarthroplasty	ChatGPT Total Hip Replacement	ChatGPT Cannulated Hip Screw	ChatGPT Dynamic Hip Screw	ChatGPT No Operation
Trauma Meeting Hemiarthroplasty	18	1	0	0	0
Trauma Meeting Total Hip Replacement	3	3	0	0	0
Trauma Meeting Cannulated Hip Screw	3	0	0	0	0
Trauma Meeting Dynamic Hip Screw	0	0	0	0	0
Trauma Meeting No Operation	2	0	0	0	0

## Discussion

This study reports the largest investigation to date of ChatGPT’s accuracy for recommending management of intracapsular NOF fractures. ChatGPT had poor initial agreement with orthopaedic consultants (κ = 0.03). After providing additional case-specific information (“training”), agreement rose significantly (κ = 0.93), but this improvement did not persist when we tested the model on a new validation cohort (κ = 0.29). These findings align with prior work showing that AI alone is generally less successful than clinicians at medical decision-making [4].

In the first round, ChatGPT rarely recommended options other than THR or hemiarthroplasty, consistent with NICE guidance that lists these procedures for intracapsular NOF fractures [10]. In routine practice, however, orthopaedic surgeons sometimes choose internal fixation (for example, cannulated hip screws or dynamic hip screws) when displacement is minimal; these techniques can be appropriate and safe for selected patients [24]. ChatGPT’s failure to recommend fixation in these scenarios may reflect strict adherence to guideline language rather than individualized clinical reasoning. If clinicians rely uncritically on such models, this tendency could promote rigid guideline adherence at the expense of individualized care and patient preferences. This is of particular importance in orthopaedics and hip fractures, where patient choice on operative and non-operative management can have life-changing consequences and is highly individualized, something which is highlighted in NICE guidance [10].

Examining the effect of the in-session adjustments we provided to ChatGPT highlights further risks of using LLMs in clinical decision-making. Those adjustments not only increased concordance with consultant decisions but also altered which patient factors the model treated as influential. After adjustment, ChatGPT’s decisions appeared to depend on age, a pattern that mirrored consultant reasoning. Although numbers were insufficient in the validation round to perform regression, the apparent pattern did not persist, and the reduction in agreement suggests the model’s weighting of factors shifted away from consultant reasoning. This pattern suggests that the model’s changed responses reflected forced alignment with the examples provided in-session rather than durable learning. To a clinician unfamiliar with LLM behavior, the transient increase in agreement could create a false impression that the model has learned from feedback, when in fact it has not.

Two related concerns underlie these risks. First, LLMs lack transparency about how they use inputs to reach outputs (a problem commonly called “lack of explainability”) [13]. Second, LLMs can develop or amplify erroneous associations that resemble bias. For example, although age correlates with factors that influence management (e.g., worse 4AT scores) [25], guidelines do not treat age alone as a determinant of management; an LLM that treats age as an independent deciding factor could introduce ethically fraught age-related bias. Other AI investigations have documented spontaneous, clinically irrelevant biases (for example, an AI video tool that assessed prostatectomy quality based on prostate size and Gleason grade rather than surgeon performance) [26]. Such opaque and spurious associations could lead to inconsistent or unsafe recommendations, particularly if junior clinicians rely on them.

Restricting AI to an educational or decision-support role (i.e., summarizing evidence and listing alternative management options while leaving the final decision to clinicians) has been proposed as one way to mitigate the explainability problem [27]. Human oversight can also help identify and correct biases; in the prostatectomy example, adding human descriptions to the training process reduced the tool’s biased judgments [26]. Even when used as a reference, however, LLMs would increase the demand for clinicians’ critical appraisal skills: clinicians must evaluate suggested options, recognize hallucinations, and apply individual patient values and circumstances. This requirement amplifies the risk associated with junior clinicians using LLMs without sufficient experience to detect errors.

In summary, transient alignment with consultant decisions, potential over-reliance on guideline text, lack of explainability, and the risk of spontaneous biases and hallucinations currently limit the safe clinical deployment of LLMs. These limitations are particularly concerning for less-experienced clinicians, who may be less able to detect flawed or inappropriate model outputs. Furthermore, it is important to highlight again that general-purpose LLMs such as ChatGPT are not designed or validated for medical decision making and currently pose significant ethical and patient safety concerns, which must be resolved before any consideration of clinical use. Education of clinicians on the safe and appropriate uses of AI through consulting guidance, such as that produced by the British Medical Association [28], is crucial to ensuring minimization of these safety concerns.

Limitations

Our study had several important limitations. First, instructing ChatGPT to follow NICE guidance may have constrained its responses. Consultants sometimes selected internal fixation (cannulated hip screws or dynamic hip screws) outside NICE recommendations; a less prescriptive prompt might have increased concordance, but could also allow the model to draw on non-recommended or lower-quality sources. Second, we could not fully control model variability between sessions. Although prompts were submitted close together, small timing gaps existed between rounds and the validation run; completing all inputs within a single session or using a fixed API/model version would reduce this source of variation. Third, when the MDT rationale was absent, the authors conferred with orthopaedic consultants to infer the reasoning behind MDT operative decisions. This approach introduces potential bias; ideally, future studies should elicit the rationale for all operative decisions at the time they are made. Fourth, small numbers of patients in certain operation or mobility categories required collapsing categories for regression analyses, which may have reduced precision and obscured associations. Finally, our in-session adjustments did not constitute true model retraining and produced only transient alignment with consultant decisions. Future work should evaluate clinically focused, validated models rather than relying on forced in-session alignment. Early examples of clinically oriented tools (for example, OpenEvidence) underline the need for rigorous clinical validation before widespread use [29,30].

## Conclusions

This study aimed to compare ChatGPT’s management recommendations for patients with intracapsular NOF fractures to decisions made by orthopaedic consultants and to evaluate which patient factors influenced those recommendations. According to our results, ChatGPT did not accurately replicate orthopaedic consultant decision-making for intracapsular NOF fractures and therefore cannot reliably advise surgeons in this setting. Although in-session adjustments increased concordance with consultant decisions, this change did not represent durable learning, as validation on an independent dataset produced agreement rates similar to those in the initial round. The model’s tendency to conform to user prompts also risks creating a false sense of security for clinicians. Furthermore, the lack of clinical validation of general-purpose LLMs for medical decision-making highlights the fundamental inappropriateness of using ChatGPT in this way. Future work should evaluate clinically focused, validated AI systems as adjuncts to, rather than replacements for, clinical judgment in orthopaedics.

## Appendices

Appendix 1

*First Prompt Template* A [AGE] year old [SEX] presents to the emergency department with an intracapsular neck of femur fracture. Their comorbidities include [COMORBIDITIES LIST]. They [WALKING STATUS]. Their 4AT score is [4AT SCORE]. Using the most recent NICE guidelines on Hip fracture management, what operation should they have to fix their intracapsular neck of femur fracture?

Notes: Age was expressed as a number in years. The options for sex and walking status are included in Table 15. Comorbidities was written as a list with commas directly from the clerking notes. 4AT score was expressed as a number.

**Table 15:** Options available for sex and walking status for first ChatGPT prompt Note: “-” denotes an intentional empty cell.

Sex	Walking Status
Male	Are independent with walking
Female	Walk by sofa surfing
-	Walk with a stick
-	Walk with two sticks
-	Walk with a frame
-	Use a wheelchair
-	Are immobile

Appendix 2

Second Prompt Template

A [AGE] year old [SEX] presents to the emergency department with an intracapsular neck of femur fracture. Their comorbidities include [COMORBIDITIES LIST]. They [WALKING STATUS]. Their 4AT score is [4AT SCORE]. ChatGPT previously recommended the patient should have a [CHAT-GPT 1ST RECOMMENDATION] to fix their intracapsular neck of femur fracture according to NICE guidelines, however several orthopaedic consultants disagree with the decision and instead think the patient should be treated with [ORTHOPAEDIC CONSULTANT RECOMMENDATION]. This is because they believe [ORTHOPAEDIC REASON]. Using the most recent NICE guidelines on Hip fracture management and the information given, would you change your management and follow the consultants’ management suggestion or would you stick with the previous ChatGPT management suggestion?

Notes: Age was expressed as a number in years. The options for sex and options for walking status are included in Table 16. Comorbidities were written as a list with commas directly from the clerking notes. 4AT score was expressed as a number. The options for Chat-GPT first recommendation and orthopaedic consultant recommendation were the same and are listed in Table 16 along with options for Orthopaedic reason.

**Table 16:** Options available for sex, walking status, ChatGPT 1st Recommendation/Orthopaedic Consultant Recommendation and orthopaedic reason for second ChatGPT prompt Note: “-” denotes an intentional empty cell.

Sex	Walking Status	ChatGPT First Recommendation/Orthopaedic Consultant Recommendation	Orthopaedic Reason
Male	Are independent with walking	Hemiarthroplasty	The patient is end of life
Female	Walk by sofa surfing	Total hip replacement	The patient is part of a clinical trial
-	Walk with a stick	Dynamic Hip Screw	The patient would prefer to have this option
-	Walk with two sticks	Cannulated Hip Screws	The fracture is not significantly displaced
-	Walk with a frame	No operation	Nil explanation in notes
-	Use a wheelchair	Internal fixation	A short anaesthetic duration is required due to the patient's comorbidities
-	Are immobile	-	The patient is fit and mobile
-	-	-	The patient has poor mobility
-	-	-	The patient has poor soft tissue coverage over the fractured hip
-	-	-	The patient is very confused and/or has severe advanced dementia
-	--	-	The patient has poor mobility and extensive significant medical comorbidities
-	-	-	The patient has extensive significant medical comorbidities
